# Rapid, complete and sustained tumour response to the TRK inhibitor larotrectinib in an infant with recurrent, chemotherapy-refractory infantile fibrosarcoma carrying the characteristic *ETV6-NTRK3* gene fusion

**DOI:** 10.1093/annonc/mdz382

**Published:** 2019-11-18

**Authors:** S S Bielack, M C Cox, M Nathrath, K Apel, C Blattmann, T Holl, R Jenewein, U Klenk, P Klothaki, P Müller-Abt, S Ortega-Lawerenz, M Reynolds, M Scheer, K Simon-Klingenstein, S Stegmaier, R Tupper, C Vokuhl, T von Kalle

**Affiliations:** 1 Pediatrics 5 (Oncology, Hematology, Immunology), Center for Pediatric, Adolescent and Women’s Medicine, Stuttgart Cancer Center, Klinikum Stuttgart – Olgahospital, Stuttgart; 2 Department of Pediatric Hematology and Oncology, University Children's Hospital Muenster, Muenster, Germany; 3 Loxo Oncology, a wholly owned subsidiary of Eli Lilly and Company, South San Francisco, USA; 4 Department of Pediatric Hematology and Oncology, Klinikum Kassel, Kassel; 5 Radiologic Institute, Center for Pediatric, Adolescent and Women’s Medicine, Stuttgart Cancer Center, Klinikum Stuttgart – Olgahospital, Stuttgart; 6 Institute for Pediatric Radiology, Klinikum Kassel, Kassel; 7 Institute of Pathology – Section Pediatric Pathology, University Hospital Schleswig-Holstein, Campus Kiel, Kiel, Germany

**Keywords:** infantile fibrosarcoma, tropomyosin receptor kinase inhibition, larotrectinib

## Abstract

**Background:**

The *ETV6-NTRK3* gene fusion is present in the majority of cases of infantile fibrosarcoma (IFS) and acts as a potent oncogenic driver. We report the very rapid, complete, and sustained response of an advanced, chemotherapy-refractory, recurrent IFS to targeted treatment with the oral tropomyosin receptor kinase (TRK) inhibitor larotrectinib.

**Patient and methods:**

A male infant born with a large congenital IFS of the tongue had the tumour surgically resected at age 4 days. Within 2 months, he developed extensive lymph node recurrence that progressed during two cycles of vincristine-doxorubicin-cyclophosphamide chemotherapy. At screening, a large right cervical mass was clinically visible. Magnetic resonance imaging (MRI) revealed bilateral cervical and axillary lymph node involvement as well as infiltration of the floor of the mouth. The largest lesion measured 5.5×4.5×4.4 cm (ca. 55 cm^3^). The patient started outpatient oral larotrectinib at 20 mg/kg twice daily at age 3.5 months.

**Results:**

After 4 days on treatment, the parents noted that the index tumour was visibly smaller and softer. The rapid tumour regression continued over the following weeks. On day 56 of treatment, the first scheduled control MRI showed the target lesion had shrunk to 1.2×1.2×0.8 cm (ca. 0.6 cm^3^), corresponding to a complete response according to the Response Evaluation Criteria In Solid Tumors version 1.1. This response was maintained over subsequent follow-up visits, and on day 112 at the second control MRI the target lymph node was completely normal. At last follow-up, the disease remained in complete remission after 16 months on larotrectinib, with negligible toxicity and no safety concerns.

**Conclusion(s):**

Selective TRK inhibition by larotrectinib offers a novel, highly specific and highly effective therapeutic option for IFS carrying the characteristic *ETV6-NTRK3* gene fusion. Its use should be considered when surgery is not feasible. (NCT02637687)

**Key Message**

Infantile fibrosarcoma (IFS) is one of few malignancies in which the majority of tumours harbour *NTRK* gene fusions. This case report describes a young infant with chemotherapy-refractory, recurrent, *ETV6-NTRK3* fusion-positive IFS who experienced rapid, pronounced and durable tumour regression during long-term treatment with larotrectinib, a selective tropomyosin receptor kinase (TRK) inhibitor.

## Background

Infantile fibrosarcoma (IFS), the most frequent sarcoma of infancy, is one of few malignancies in which the vast majority of tumours carry *NTRK* rearrangements [1–3], with the *ETV6-NTRK3* gene fusion occurring in about 70% of cases of IFS [4]. While surgery alone or in combination with chemotherapy is often curative, there are cases when such treatment is either unsuccessful or would lead to major mutilation and disability [5, 6]. Previously published cases have demonstrated the rapid and robust efficacy of the selective tropomyosin receptor kinase (TRK) inhibitor larotrectinib in patients with IFS, including when used as neoadjuvant therapy. However, these reports did not provide data on the long-term administration of this agent [7, 8]. Here, we present the case of a young infant with chemotherapy-refractory, recurrent IFS who experienced rapid, pronounced and durable tumour regression during long-term treatment with larotrectinib.

## Patient and methods

**Figure 1.**
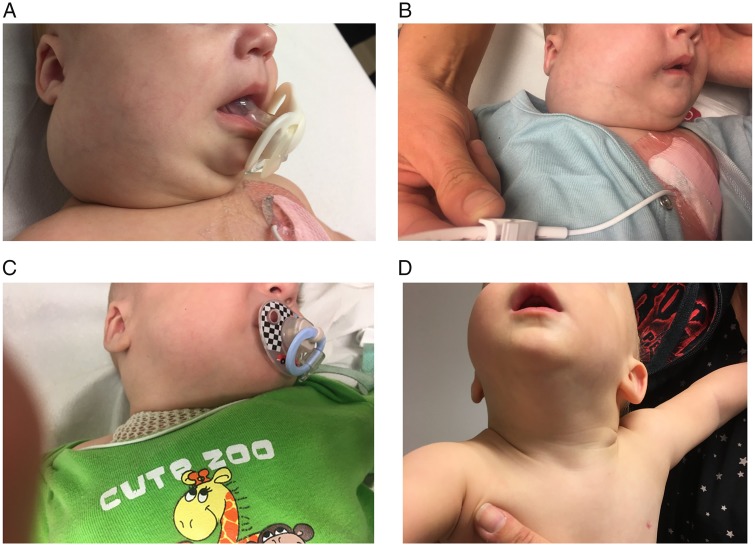
Clinical impression of the right-sided cervical mass (A) before initiation of larotrectinib, (B) at week 3 of treatment, (C) at week 9 of treatment and (D) at week 25 of treatment.

This otherwise healthy male infant was born with a large congenital IFS that was located on the anterior portion of the tongue and protruded from the mouth. The tumour was surgically resected with close margins (R1/RX) when the patient was aged 4 days. Pathological workup, including break-apart fluorescence *in situ* hybridisation, revealed IFS with *ETV6*-breakage, and PCR confirmed the presence of the characteristic *ETV6-NTRK3* rearrangement. At age 7 weeks, the parents noted a rapidly growing mass of the right cervical region. Contrast-enhanced magnetic resonance imaging (MRI) revealed a bilateral cervical lymph node recurrence. The largest, right-sided cervical mass measured 4.5 × 4.2 × 2.6 cm (ca. 26 cm³) and there was a contralateral pathological lymph node measuring 2.3 × 1.3 × 1.7 cm. A contrast-enhancing mass of the right submandibular region measuring 2.5 × 1.7 × 1.6 cm infiltrated the musculature of the floor of the mouth. In addition, left axillary lymph node involvement was suspected. Given the extent of regional tumour involvement, surgery was not considered feasible. A trial of cytotoxic chemotherapy was therefore initiated, and the patient received two cycles of cyclophosphamide-doxorubicin-vincristine, but the tumour did not respond. Instead, the clinical impression was progression of the right-sided cervical mass (Figure 1A).

**Figure 2.**
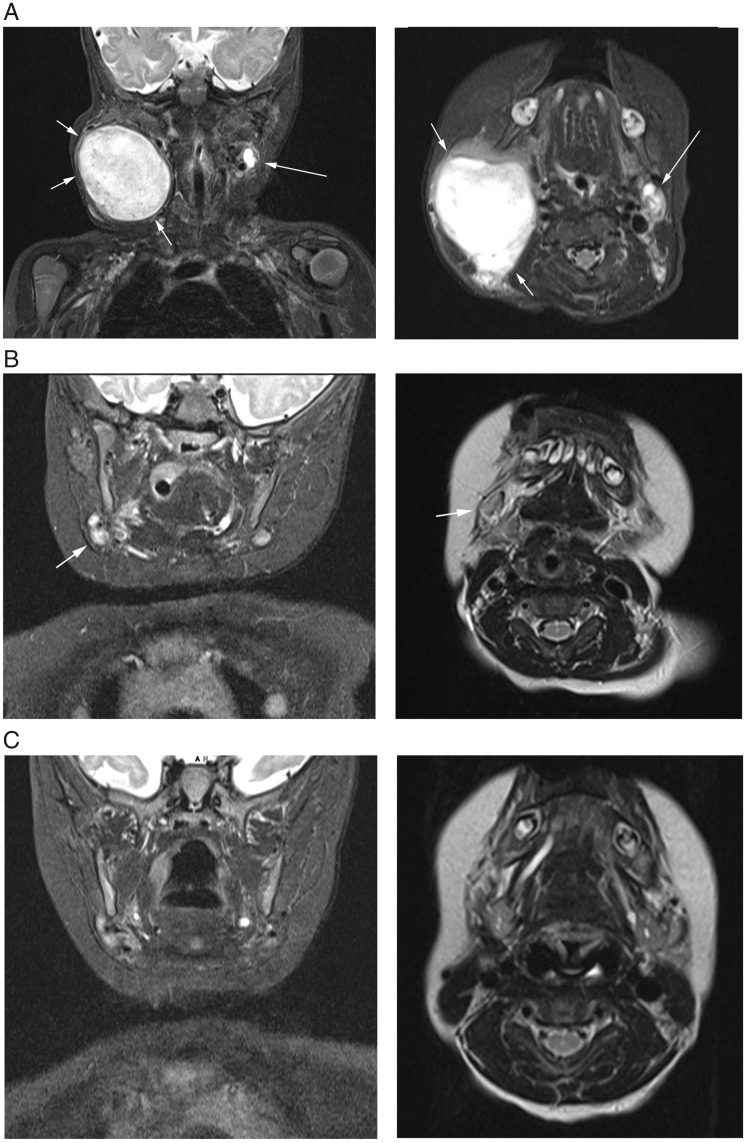
Visualisation of the largest diameters of the right cervical lesion upon coronal and planar magnetic resonance imaging (A) at screening, (B) on day 56 of treatment and (C) on day 112 of treatment.

Consequently, the patient was invited to participate in a multicentre, open-label, phase I/II study investigating the efficacy and safety of larotrectinib for the treatment of advanced paediatric solid or primary central nervous system (CNS) tumours (NCT02637687) [9]. After informed consent was obtained, the patient was screened for enrolment and found to be eligible. Screening MRI confirmed disease progression, with the right-sided cervical index lesion now measuring 5.5 × 4.5 × 4.4 cm (ca. 55 cm³; Figure 2A). Outpatient treatment with oral larotrectinib at a dose of 100 mg/m^2^ body surface area twice a day, corresponding to 1.5 ml larotrectinib 20 mg/ml solution twice daily, was initiated at age 3.5 months (cycle 1 day 1; C1D1).

## Results

The parents noticed a change in tumour consistency within 4 days, which was confirmed during the next clinic visit at C1D8 when the index lesion appeared softer, more mobile upon palpation and reduced in size. The rapid tumour regression continued over the following days and weeks (Figure 1B–D). At the first scheduled control MRI carried out on day 56 of larotrectinib treatment (C3D1), the only abnormality remaining was a solitary, slightly enlarged right cervical lymph node which measured 1.2 × 1.2 × 0.8 cm (ca. 0.6 cm³; Figure 2B). According to the Response Evaluation Criteria in Solid Tumours version 1.1 for lymph node response, in which target nodes are measured in the axis perpendicular to the longest diameter [10, 11], this corresponded to a complete response (CR). CR was maintained at the second control MRI on C5D1, with the previously described lymph node now completely normal (Figure 2C), and at last follow-up on C18D1 (16 months after initiation of larotrectinib).

Except for a brief period of grade 3 increased alkaline phosphatase, which was considered unrelated to larotrectinib, the patient experienced no adverse events of grade 3 or higher (coded according to the National Cancer Institute’s Common Terminology Criteria for Adverse Events version 4.3). His growth was consistent with his percentiles and he met his developmental milestones, learning to walk at age 11 months. Vaccinations were carried out as recommended by the German Steady Vaccination Committee without severe or unexpected complications (1 day of fever following the measles-mumps-rubella vaccine).

## Discussion

The presented IFS case illustrates that targeted therapy of TRK fusion cancer with the selective TRK inhibitor larotrectinib can result in rapid, dramatic responses even after failure of chemotherapy. The majority of IFS cases carry *NTRK* gene fusions, similar to a number of other malignancies, including mesoblastic nephroma, secretory breast cancer and the mammary analogue secretory carcinoma of the salivary glands [1, 12]. This characteristic gene fusion renders them potentially vulnerable to treatment with TRK inhibitors, such as larotrectinib, which received accelerated US Food and Drug Administration (FDA) approval based on data from three multicentre, open-label, single-arm clinical trials [9, 13, 14].* This is only the second tissue-agnostic drug approved by the FDA for the treatment of cancer [14]. Larotrectinib has demonstrated durable responses in a wide variety of *NTRK* gene fusion-positive tumours in both children and adults [9, 15, 16]. Of note, all eight patients with *NTRK* gene fusion-positive IFS treated in the phase I portion of the larotrectinib paediatric trial responded to treatment: six patients with a partial response and two patients with a CR [8, 15]. Five of the patients with partial response underwent surgical resection, with R0 in three patients, R1 in one patient and R2 in one patient. At the time of surgical resection, two patients had complete pathological responses.

Larotrectinib is generally well tolerated in both adults and children [9, 15]. To date, most adverse events reported have been grade 1 or 2, with no grade 4 or 5 treatment-related adverse events observed [9, 15]. However, although acute toxicities seen with larotrectinib are usually minor and rarely cause treatment interruption or dose reduction, little is known about the long-term effects of this drug, particularly when administered over prolonged periods to young infants and children who are still growing. It is therefore reassuring that our patient seems to have developed appropriately for his age over the 11 months that he received larotrectinib treatment. It is also of note that he was vaccinated with toxoid, inactivated and live-attenuated vaccines with no unexpected side effects. It remains to be seen whether his development and that of other young children will remain uneventful if larotrectinib treatment is continued for many years. In normal physiology, TRKs are involved in the neuronal development, function and maintenance of the CNS and peripheral nervous system, with important roles in neuronal cell survival, morphology and differentiation [17, 18], as well as in the regulation of sensation, movement, behaviour and cognition [19]. As the long-term effects of TRK inhibition on the CNS and peripheral nervous system are currently unknown, it may be advisable to include neurophysiological and neuropsychological testing in the long-term follow-up of patients treated with TRK inhibitors, particularly when they have been exposed to these agents as infants or young children. The potential for TRK inhibition to be used in paediatric upfront settings, without prior chemotherapy, may only be fully exploited once studies confirm the long-term safety of this approach.

Even with the limited clinical use of larotrectinib to date, remissions lasting more than 2 years have already been reported for patients whose inoperable localised or metastatic tumours responded to the drug [9, 15]. However, the proportion of tumours that will permanently respond to continued TRK inhibition is unknown as secondary resistance has been observed in a minority of patients treated with larotrectinib, possibly resulting from mutations altering the kinase domain of TRK [9, 20]. Research is ongoing to elucidate resistance mechanisms to TRK inhibitors and identify strategies for overcoming resistance mutations [20]. In addition, too few patients with responding malignancies have terminated larotrectinib in the absence of complete surgery to determine whether and when treatment may be interrupted, or even stopped, when enduring drug-induced remission is achieved [7]. Hence, further research is warranted to determine the role of TRK inhibition in the long-term management of *NTRK* gene fusion cancers.

## Conclusion

In summary, larotrectinib offers a novel, well-tolerated and often highly effective treatment for patients with IFS and other *NTRK* gene fusion-positive tumours. The present case report is consistent with previous evidence and provides a graphic example of a dramatic clinical and radiological response in an infant with refractory infantile fibrosarcoma. 

## Footnotes

* Note added in proof: The European Medicines Agency granted marketing authorisation for larotrectinib on 23 September 2019 as monotherapy for the treatment of adult and paediatric patients with solid tumours that display a neurotrophic tyrosine receptor kinase (*NTRK*) gene fusion, and who have disease that is locally advanced, metastatic or where surgical resection is likely to result in severe morbidity, and who have no satisfactory treatment options.
